# The Mechanism of NMDA Receptor Hyperexcitation in High Pressure Helium and Hyperbaric Oxygen

**DOI:** 10.3389/fphys.2020.01057

**Published:** 2020-08-25

**Authors:** Alice Bliznyuk, Michael Hollmann, Yoram Grossman

**Affiliations:** ^1^Department of Physiology and Cell Biology, The Zlotowski Center for Neuroscience, Faculty of Health Sciences, Ben-Gurion University of the Negev, Beersheba, Israel; ^2^Israel Naval Medical Institute, Haifa, Israel; ^3^Receptor Biochemistry, Faculty of Chemistry and Biochemistry, Ruhr-University Bochum, Bochum, Germany

**Keywords:** NMDAR, HPNS, GluN2A, high pressure, zinc, HBO, O_2_ toxicity, *Xenopus laevis* oocytes

## Abstract

Professional divers exposed to pressures greater than 1.1 MPa may suffer from the high pressure neurological syndrome (HPNS). Divers who use closed-circuit breathing apparatus face the risk of CNS hyperbaric oxygen toxicity (HBOTox). Both syndromes are characterized by reversible CNS hyperexcitability, accompanied by cognitive and motor deficits. Previous studies have demonstrated that the hyperexcitability of HPNS is induced mainly by NMDA receptors (NMDARs). In our recent studies, we demonstrated that the response of NMDARs containing GluN1 + GluN2A subunits was increased by up to 50% at high pressure (HP) He, whereas GluN1 + GluN2B NMDARs response was not affected under similar conditions. Our aim was to compare the responses of both types of NMDARs under HBOTox conditions to those of HP He and to reveal their possible underlying molecular mechanism(s). The two combinations of NMDARs were expressed in *Xenopus laevis* oocytes, placed in a pressure chamber, voltage-clamped, and their currents were tested at 0.1 (control) −0.54 MPa 100% O_2_ or 0.1–5.1 MPa He pressures. We show, for the first time, that NMDARs containing the GluN2A subunit exhibit increased responses in 100% O_2_ at a pressure of 0.54 MPa, similar to those observed in 5.1 MPa He. In contrast, the GluN1 + GluN2B response is not sensitive to either condition. We discovered that neither condition produced statistically significant changes in the voltage-dependent Mg^2+^ inhibition of the response. The averaged IC_50_ remained the same, but a higher [Mg^2+^]_*o*_ was required to restore the current to its control value. The application of TPEN, a Zn^2+^ chelator, in control, HP He and HBOTox conditions, revealed that the increase in GluN1 + GluN2A current is associated with the removal of the high-affinity voltage-independent Zn^2+^ inhibition of the receptor. We propose that HPNS and HBOTox may share a common mechanism, namely removal of Zn^2+^ from its specific binding site on the N-terminal domain of the GluN2A subunit, which increases the pore input-conductance and produces larger currents and consequently a hyperexcitation.

## Introduction

N-methyl-D-aspartate receptors (NMDARs) are members of the ionotropic glutamate receptor (iGluR) family, which also includes AMPA and kainate receptors. iGluRs convert transient glutamate release from presynaptic vesicles into post-synaptic neuronal excitation at synapses. This excitatory neurotransmission is one of the fundamental mechanisms for the correct development and function of the mammalian brain. Calcium influx (a considerable fraction of the receptor current) through open NMDARs is pivotal to cellular synaptic transmission and plasticity during learning and memory (Traynelis et al., 2010). NMDAR calcium flow is controlled by multiple patterns of heterotetramerization of GluN1 subunits (eight GluN1-1a to -4a and GluN1-1b to -4b subunit variants resulting from alternative RNA splicing (Dingledine et al., 1999), GluN2 (encoded by four different genes, 2A to 2D) and GluN3 subunits (two different genes 3A–3B). Each NMDAR subunit contains a large extracellular N-terminal domain (NTD), a ligand-binding domain (LBD), a transmembrane domain (TMD) that has three membrane-spanning domains, a re-entry loop that forms the pore-lining region (membrane domain 2), and an intracellular C-terminal domain (CTD) (Traynelis et al., 2010). NMDARs containing GluN2A or GluN2B have a high-conductance channel opening, and are sensitive to voltage-dependent block by Mg^2+^. The magnesium binding site (containing Asn residues, N and N + 1 sites) is located at the channel pore in the TMD of the receptor, and is responsible for the voltage-dependent block (Wollmuth et al., 1998; Tu and Kuo, 2015). Structural studies of the GluN2A subunit have revealed a distinctive Zn^2+^ binding site in the external NTD of the receptor that contains four residues: H44, H128, E266, and D282. The receptor channel is allosterically inhibited by nM concentrations of Zn^2+^, which is termed high-affinity voltage-independent Zn^2+^ inhibition (Paoletti et al., 1997; Romero-Hernandez et al., 2016). Hyper- or hypo-activation of NMDARs has been implicated in neurological disorders such as epilepsy, stroke, schizophrenia, and Alzheimer’s disease (Paoletti et al., 2013).

Hyperactivation of NMDARs may also be associated with two known major diving-related central nervous system (CNS) syndromes: high pressure neurological syndrome (HPNS), and CNS hyperbaric oxygen toxicity (HBOTox). Professional divers exposed to pressures greater than 1.1 MPa usually suffer from HPNS (Halsey, 1982; Tibbles and Edelsberg, 1996; Bennett and Rostain, 2003; Grossman et al., 2010). HPNS is characterized by reversible CNS hyperexcitability, accompanied by cognitive and motor deficits. Previous studies have demonstrated that the hyperexcitability of HPNS is induced mainly by NMDARs (Fagni et al., 1985, 1987; Zinebi et al., 1988, 1990; Daniels and Grossman, 2003; Mor and Grossman, 2010). In contrast, other members of the iGluR family play a very small part in inducing CNS hyperexcitation at high pressure (HP). The AMPA receptor did not exhibit a significant response to HP (Pearce et al., 1994), and kainate receptors were totally unaffected by pressure (Shelton et al., 1993). Other amino acid-activated ionotropic receptors, such as GABA receptors, are also insensitive to HP (Roberts et al., 1996). The maximal response of glycine receptors remained unchanged, although the IC_50_ was considerably elevated at HP (Shelton et al., 1993). Electrophysiological studies of rat brain slices in HP He showed a significant increase in the synaptic NMDAR response, followed by post-synaptic excitability changes (Mor and Grossman, 2006, 2007). In addition, it was demonstrated that the well-known voltage-dependent inhibition by Mg^2+^ decreases twofold under HP He conditions (Mor and Grossman, 2010). A study of oocytes expressing different subtypes of the receptor found that only NMDARs containing the GluN2A subunit exhibit an increase in current in HP He, whereas GluN1 in combination with other GluN2 subunits produced only minor inhibition or no change in current at all (Mor et al., 2012). The extent of the increase or decrease in current depends on the presence of the GluN1 subunit variant in the receptor (Bliznyuk et al., 2015, 2016, 2019a). The GluN2A and GluN2B subunits are the most abundant in the adult CNS, and generate large currents. Because there is no change in the current of GluN1 + GluN2B receptors, whereas GluN1 + GluN2A receptor responses increase by up to 50%, we may consider GluN2A as the only subunit responsible for the CNS hyperexcitability induced by HP He. All of the above investigations suggest that HP may alter the receptor’s 3D conformation. Recreational, commercial and combat divers who use closed-circuit breathing apparatus (100% O_2_ or a mixture of O_2_ and one or more inert gases) at O_2_ pressures greater than 0.2 MPa, face the risk of HBOTox, whose major symptoms are also reversible CNS hyperexcitability, convulsions and loss of conscience (Butler and Thalmann, 1986; Acott, 1999; Clark and Thom, 2003; Dean et al., 2003). A study *in vivo* showed that parenteral Mg^2+^ (MgSO_4_) administration may provide partial protection against CNS HBOTox by delaying the appearance of seizures (Katz et al., 1990). A similar risk has to be considered for patients and attendants during HBO therapy (HBOT) over that pressure range. The general mechanism behind HBOTox is most probably oxidative stress, namely the presence of high levels of reactive oxygen species (ROS) that may damage any of the human biological systems (Dean et al., 2003). However, little is known regarding specific molecular mechanism(s) of CNS HBOTox. The purpose of the present study was to examine whether the response of NMDARs containing the GluN2A subunit increases in 100% O_2_ at 0.54 MPa in a similar fashion to that observed in He at 5.1 MPa. We hypothesized that the GluN2B subunit response in HBO would remain unaffected as it did in HP He, and that the mechanism underlying the increase in the GluN2A subunit current in both HBO and HP He may be the removal of the basal high-affinity voltage-independent Zn^2+^ inhibition of the receptor.

## Materials and Methods

### Two Electrode Voltage Clamp

#### Oocyte Preparation

Stage V and VI oocytes were surgically removed from the ovaries of *Xenopus laevis* anesthetized with 1.5 g/L ethyl 3-aminobenzoate methanesulfonate salt (Sigma-Aldrich, Israel). Oocytes were prepared and maintained at 18°C in ND-96 solution containing NaCl 96 mM, KCl 2 mM, MgCl_2_ 1 mM, CaCl_2_ 1.8 mM, sodium pyruvate 2.5 mM, HEPES 5 mM (Amresco LLC, Solon, OH, United States), PEN/STREP 10 mg/ml, and gentamicin 50 μg/ml, adjusted to pH 7.5. The incisions were closed by absorbable sutures and animals were returned to the tank. Surgery was performed according to the guidelines laid down by the Ben-Gurion University of the Negev ethics committee for the care and use of animals for experimental work (IL-69-12-2011). Within 24 h of surgery, oocytes were injected with one of the two newly synthesized GluN1-1a splice variant cRNAs 5 ng, and either GluN2A or GluN2B subunit cRNA 5 ng (see text for the specific experiments) using a nanoliter injector (World Precision Instruments, Sarasota, FL, United States). All cRNAs were produced by Prof. M. Hollmann’s laboratory (Ruhr University, Bochum, Germany). The NMDAR cDNA GenBank accession numbers are: GluN1-1a: U08261; GluN2A: AF001423; and GluN2B: U11419. All NMDAR subtypes were successfully expressed at the oocyte plasma membrane that was tested by functionality test, a current recording at a control conditions (see section “NMDAR Current Recordings”).

#### Pressure, Compression, and Decompression

The experimental setup, pressure chamber, perfusion system, and compression in He or O_2_, have been described in detail previously (Mor and Grossman, 2006). Briefly, experiments were conducted in a pressure chamber (Canty Associates, NY, United States). HP was attained by compression with He or 100% O_2_ to an experimental pressure range of 0.1–5.1 MPa for He and 0.1–0.54 MPa for O2. This range of HBO testing was based on the following rationale: Humans HBOtox threshold is considered to be at 0.2 MPa. Therefore, this is the normal maximal pressure for HBO therapy. Divers using 100% O2 are not allowed to dive deeper than 10 m (8 m = 0.18 MPa as safety limit). In the case of rebreather diving, this limit should be kept dynamically upon diving with any gas mixture and various encountered ambient pressures. However, rodents show resistance to HBOtox up to 0.5–0.6 MPA of O2. Since many of the experiments on HBOTox were performed on rats or mice, and our subunits were derived from gene sequencing of rat, we chose to expose our NMDARs to HBO up to 0.54 MPa. The rates of compression/decompression varied between 0.05 and 0.5 MPa/min. To avoid transient effects of pressure (Grossman and Kendig, 1984), recordings were taken at a strictly controlled ambient temperature (25 ± 1°C). The typical time to reach 5.1 MPa He and stable temperature conditions was 20–25 min, including the time required for stabilization of temperature transients of ± 1–3°C during compression and decompression. This pressure step is a routine procedure consistently used in our laboratory to demonstrate the effects of HP. Recovery at 0.1 MPa was always attempted.

The time required to reach 0.54 MPa O_2_ in stable conditions was 5–10 min. The first recording was made 15 min after compression began to ensure the appearance of HBOTox.

#### NMDAR Current Recordings

After incubation for 3–4 days (the time needed for NMDAR expression on the oocyte membrane), individual oocytes were placed in a custom-designed recording bath and inserted in the pressure chamber. They were superfused at a constant velocity of 7–8 ml/min with frog physiological (recording) solution containing NaCl 90 mM, KCl 1 mM, BaCl_2_ 1.5 mM (substituted for Ca^2+^ as a charge carrier), HEPES 10 mM (Amresco LLC, Solon, OH, United States), and zero added Mg^2+^ in order to eliminate the known physiological Mg^2+^ blockade of NMDARs. The solutions, saturated with air for the HP He experiments and 100% O_2_ for the HBO experiments, were continuously introduced into the pressure chamber using a high-pressure pump (“minipump,” LDC Analytical Inc., Riviera Beach, FL, United States). Testing of each NMDAR subtype included at least two separate batches of oocytes obtained from different frogs.

Oocytes were voltage-clamped at -70 mV employing the two-electrode voltage-clamp technique, using a GeneClamp 500-amplifier (Molecular Devices, Axon Instruments Inc., CA, United States). The co-agonists glutamate 100 μM and glycine 10 μM (Sigma, Israel) were added to the physiological solution and applied in the course of a 20-s exposure for measuring currents through the receptors (Bliznyuk et al., 2015). Leak (baseline) currents are subject to change during compression and decompression procedures, and they sometimes differ under hyperbaric and control conditions (0.1 MPa). Nevertheless, under constant pressure, temperature, solution flow rate, pH, etc., leak currents are stable and may thus be subtracted from the NMDAR responses. The oocyte membrane holding potential (-70 mV) was monitored continuously; deviations of up to ± 1 mV were accepted. Time control protocols carried out for 2–3 h in previous studies conducted by our laboratory showed oocyte stability under control, HP, and decompression conditions (Mor et al., 2012; Aviner et al., 2014; Bliznyuk et al., 2015).

To verify our hypothesis that voltage-dependent inhibition by Mg^2+^ decreases under HP conditions, different [Mg^2+^]_*o*_ were added to the recording solution in the form MgCl_2_, and were tested under control and HP conditions.

Although all experiments were carried out in nominally zero Zn^2+^, some contamination of heavy ions may have been present. To test the hypothesis that voltage-independent inhibition by Zn^2+^ may be removed under pressure conditions, the GluN1-1a + GluN2A receptor subtype was expressed in oocytes and ionic currents were measured in Ba^2+^ solution ([Mg^2+^]_*o*_ = 0). The effect of 1 μM TPEN (N,N,N’,N’-tetrakis (2-pyridinylmethyl)-1,2-ethanediamine) (Biotium, Fremont, CA, United States), a Zn^2+^ chelator, was tested under control conditions (0.1 MPa), in 100% O_2_ at 0.54 MPa and HP He at 5.1 MPa.

### Experimental Data Collection and Statistical Analysis

In each experiment, the same oocyte was subjected to both control and HP He or HBO conditions. pH, temperature, solute concentrations, flow rate, and agonists concentrations remained the same, the only difference being the exposure to different pressures in He or O_2_. At each pressure step, the same agonist application was repeated up to 5 times and at least 2–3 highly similar current traces were acquired under the same conditions and averaged. In some cases where the responses were unstable due to increased leak current or large difference in maximal current the oocyte was later excluded from the study. In case that under normal conditions we could not detect any current response to several applications of the receptor’s agonists, we concluded that the targeted NMDAR was not expressed on the particular oocyte membrane and the oocyte was discarded and was not compressed.

Because each experimental oocyte served as its own control, we commonly used paired sample Student *t*-test analyses. The results of maximal current amplitude measurements are expressed as mean amplitude ± 1 standard error of the mean (SEM); *n* denotes the number of successful experiments (number of oocytes) for each experimental protocol. The degree of significance was denoted by the values of *p*. Results were considered statistically significant when *p* < 0.05.

Graphical representations were made using OriginLab software (OriginLab, Northampton, MA, United States) or MatLab^®^.

Calculations of the dose-response curve fit were made using the OriginLab software function “Growth/Sigmoidal/DoseResp”:

$$y=A\,1+\frac{A\,2-A\,1}{1+10^{\left(\mathrm{Logx}\,0-x\right)\,p}}$$

*A1* = bottom asymptote, *A2* = top asymptote, *Logx0* = center, *p* = Hill slope, *Logx0* = −5.0 (vary), *p* = 1.0 (vary).

## Results

### HBO Increases NMDAR Subtype GluN1-1a + GluN2A Currents

**FIGURE 1:**
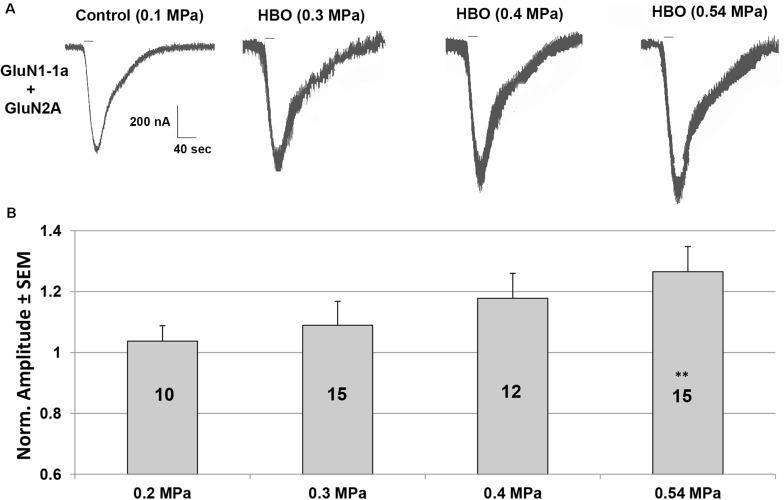
Current amplitudes of the GluN1-1a + GluN2A subtype in HBO at different pressures. **(A)** An example of HBO effects on GluN1-1a + GluN2A current response. All pressure steps were applied to the same oocyte. The applied agonists were glutamate (100 μM) and glycine (10 μM) with no [Mg^2+^]_*o*_ added. The 20 s agonists application time is indicated by horizontal bars. **(B)** Quantitative analysis of the NMDAR currents. Responses were normalized to the control value at 0.1 MPa (when the recording solution is saturated with 100% O_2_) for each tested oocyte (indicated by numbers in the bars). Oocytes were exposed to 2–4 increased pressure steps. ^∗∗^*p* < 0.01.

An increased response in the GluN1-1a + GluN2A subtype was observed under elevated HBO. Examples of the current responses are shown in Figure 1A. A detailed statistical analysis of the NMDAR responses (Figure 1B) revealed a good linear correlation between the increase in current response and pressure (*y* = 0.0668× + 0.9004, *R*^2^ = 0.9918), although only the response at 0.54 MPa was statistically significant.

Experiments on GluN1-1a + GluN2B subtype indicated that there is no change in the currents at 0.54 MPa HBO (−8.58 ± 5.41%, *n* = 7, *p* = 0.23; Figure 5A), as also reported for HP He (Mor et al., 2012).

### Voltage-Dependent Mg^2 +^ Inhibition of NMDAR Subtype GluN1-1a + GluN2A Currents Is Not Affected by HP He and HBO

**FIGURE 2:**
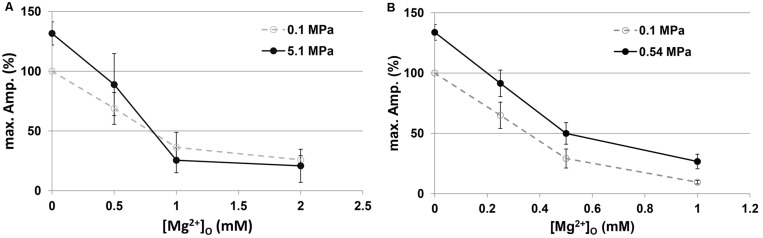
Current amplitudes of the GluN1-1a + GluN2A subtype at different [Mg^2+^]_*o*_. Currents were normalized (%) to the [Mg^2+^]_*o*_ = 0 response at 0.1 MPa. **(A)** Compression with He (*n* = 8). **(B)** Compression with 100% O_2_ (*n* = 11).

**FIGURE 3:**
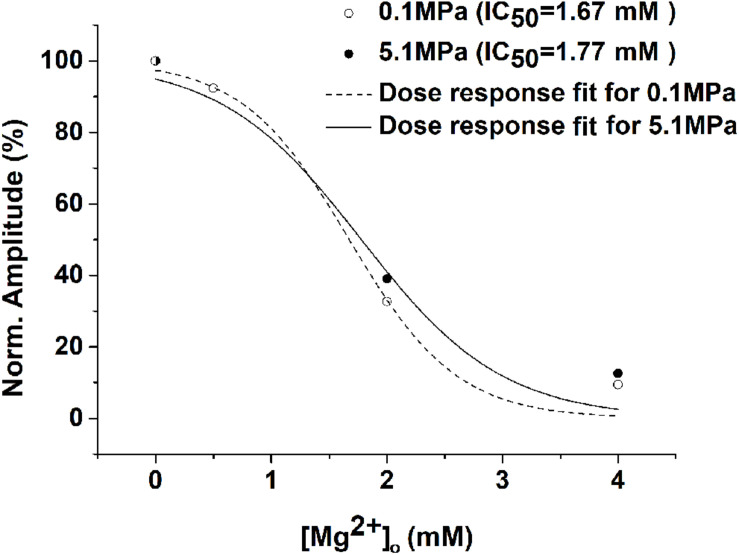
Example of the HP He effect on the Mg^2+^ dose-response curve. There were no significant changes in the dose-response curve and the IC_50_ for the GluN1-1a + GluN2A subtype (measurements were performed on the same oocyte under control and HP He conditions). Current amplitudes of the receptor were normalized to its [Mg^2+^]_*o*_ = 0 response, and dose-response curve fit was performed for each experiment.

As expected from previous experiments (Mor and Grossman, 2010) and the present study (Figure 1), HP He and HBO increased the GluN1-1a + GluN2A subtype response by 30–35% in the absence of added [Mg^2+^]_*o*_ (Figure 2). The addition of Mg^2+^ to the solution (in the form MgCl_2_) decreased the currents under control and pressure conditions. However, the averaged IC_50_ in control (0.98 ± 0.17 mM, *n* = 8) and HP He experiments (0.99 ± 0.19 mM, *n* = 8; Figure 2A) exhibited no statistically significant difference (see example, Figure 3). This was also the case for averaged IC_50_ in control (0.39 ± 0.06 mM, *n* = 11) and HBO experiments (0.40 ± 0.05 mM, *n* = 11; Figure 2B).

### Voltage-Independent Zn^2 +^ Inhibition of NMDAR Subtype GluN1-1a + GluN2A Currents Is Removed in HP He and HBO

**FIGURE 4:**
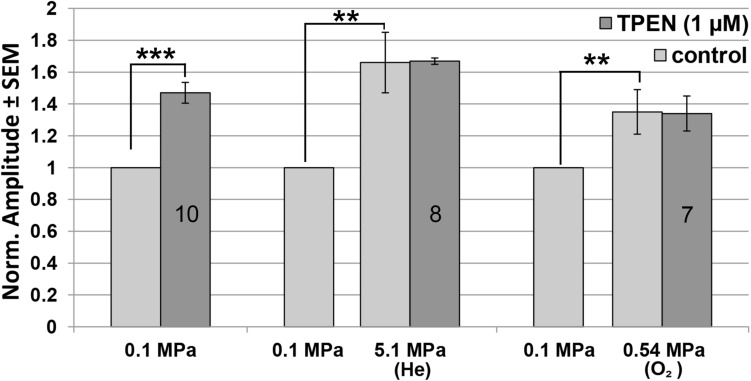
Quantitative analysis of GluN1-1a + GluN2A current changes in HP He and HBO with and without TPEN. Current amplitude is expressed as mean ± SEM in control (0.1 MPa), 0.54 MPa O_2_ and 5.1 MPa He, with 0 μM or 1 μM TPEN. Splice variant GluN1-1a was co-expressed with the GluN2A subunit. Numbers in the bars indicate number of oocytes tested. **p < 0.005, ***p < 0.001.

The addition of TPEN at 0.1 MPa increased the GluN1-1a + GluN2A subtype currents by 47.6 ± 6.5% (*n* = 10, *p* < 0.001; Figure 4), whereas administration of TPEN in 0.54 MPa O_2_ or 5.1 MPa He elicited no significant change over and above the preceding pressure effect: −0.43 ± 1.12% (*n* = 7, *p* = 0.56) and 0.74 ± 2.36% (*n* = 8, *p* = 0.79), respectively. Compression with O_2_ increased the current by 35.56 ± 14.04% (*n* = 7, *p* = 0.005), and with He by 66.86 ± 18.68% (*n* = 8, *p* = 0.005).

**FIGURE 5:**
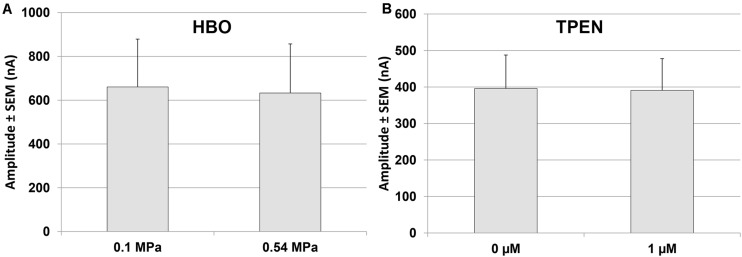
Quantitative analysis of GluN1-1a + GluN2B currents in HBO and with TPEN. **(A)** Current amplitude expressed as mean ± SEM in control (0.1 MPa) and 0.54 MPa O_2_ (*n* = 7). **(B)** Current amplitude expressed as mean ± SEM with 0 μM or 1 μM TPEN in control (0.1 MPa) (*n* = 5). No statistically significant difference was found for either experiment.

Experiments were also performed on the GluN1-1a + GluN2B subtype at the control pressure of 0.1 MPa. The results indicated that TPEN does not induce any change in the current amplitude (0.78 ± 3.27%, *n* = 5, *p* = 0.78; Figure 5B). This was to be expected, because GluN2B does not have the Zn^2+^ voltage-independent inhibition site.

## Discussion

The results of the present study show for the first time that HBO at 0.54 MPa significantly increases the NMDAR current response of the GluN1-1a + GluN2A receptor subtype. This may be one of the major underlying mechanisms of the EEG hyperexcitation typical of HBOTox. However, we were unable to demonstrate a clear threshold for the effect, which is less pronounced at 0.1–0.2 MPa. This would suggest little involvement of NMDARs in any HBO response within that pressure range, although there was a good linear correlation between the increase in current response and O_2_ pressure (*y* = 0.0668× + 0.9004, *R*^2^ = 0.9918).

It is known that prolonged exposure to low levels of HBO, such as pressures between 0.18–0.2 MPa used regularly for HBOT, may cause CNS or pulmonary HBOTox (Butler and Thalmann, 1986; Acott, 1999; Clark and Thom, 2003; Dean et al., 2003). Although we failed to demonstrate a statistically significant increase in the currents at 0.2 MPa after exposure for 15–30 min, one may nevertheless speculate that even a small increase of ∼3% over a few hours may cause significant reversible CNS hyperexcitability. At lower O_2_ partial pressures (35–40% O_2_ at 0.12–0.13 MPa), which have been proposed as “mild HBO treatment” having some therapeutic benefit (Ishihara, 2019), we would not expect any change in NMDAR activity.

We have previously demonstrated that NMDARs containing the GluN2A subunit are the only ones to increase their current/input-conductance under HP He conditions [see (Bliznyuk et al., 2015) for a discussion of the correlation between current and input-conductance increase]. In the present study, we show for the first time that this NMDAR subtype responds in a similar fashion to HBO at 0.54 MPa, a finding that was somewhat surprising, but which suggests a possible shared mechanism. Although all experiments were carried out in nominally zero Zn^2+^, we realized that contamination of Zn^2+^ in the nM range may have been present in our recording solution. The elimination of Zn^2+^ by TPEN at the control pressure of 0.1 MPa increased the NMDAR current by ∼50%, revealing the presence of strong resting voltage-independent Zn^2+^ inhibition of the GluN1-1a + GluN2A receptor. Most importantly, the addition of TPEN in both HBO and HP He failed to alter the currents. The lack of any additional response to TPEN strongly supports the notion that voltage-independent inhibition by Zn^2+^ had already been removed under both conditions. The absence of any effect of HP He and HBO on the GluN1-1a + GluN2B NMDAR, which lacks the Zn^2+^ voltage-independent inhibition site, greatly strengthens this hypothesis. However, the increase in current at 0.54 MPa HBO was only ∼35%, whereas at 5.1 MPa HP He this was ∼67%. It appears that the removal of inhibition by Zn^2+^ is only part of the mechanism responsible for the HP increase in currents.

In both He and O_2_, there was no statistically significant change in inhibition by Mg^2+^. The averaged IC_50_ remained the same, but higher [Mg^2+^]_*o*_ was required to restore the increase in current to control values (Figure 2). This may explain the success of MgSO_4_ treatment in significantly prolonging the latency to EEG manifestations of CNS HBOTox in rats (Katz et al., 1990), which led the authors to suggest MgSO_4_ pretreatment for the prevention of HBOTox in divers. It is important to note that the Mg^2+^ effect may also be due to its capacity for blocking synaptic release, as is evident from the study of Mor and Grossman (2010), who showed that voltage-dependent Mg^2+^ inhibition of the NMDAR-dependent synaptic response is only half as effective in HP He.

The present study and our previous investigations (Bliznyuk et al., 2015, 2016, 2019b) have demonstrated that aggregate formation, modified stoichiometry, alterations in glutamate and glycine affinity, and an increase in the number of receptors exposed on the membrane, cannot explain the increase in current (input-conductance) of NMDAR containing the GluN2A subunit. Yet our molecular dynamics simulations (Bliznyuk et al., 2019a) have indeed suggested that hydrostatic pressure and compression with He may cause alterations in NMDAR protein conformation. This, together with our molecular and electrophysiological data, leads us to suggest that part of the mechanism underlying the HBO- and HP He-induced increase in the current of NMDARs containing the GluN2A subunit is the removal of Zn^2+^ from its specific binding site on the NTD. Alterations of this kind will propagate to the LBD layer and the ion channel region in the TMD, resulting in an increase in pore input-conductance. However, high pressure may alter not only the Zn^2+^ binding site, but also other unknown locations which can potentially modify receptor activity. Additional molecular dynamics simulations are therefore required to establish the predicted change in the Zn^2+^ binding site, which may also explain the different responses of NMDAR proteins to pressure as mediated by various gas molecules. We are tempted to speculate that in the future, parenteral administration of Mg^2+^ and Zn^2+^ may be used as a means of neuroprotection to prevent HBOTox and HPNS in divers.

## Data Availability Statement

All datasets presented in this study are included in the article/supplementary material.

## Ethics Statement

The animal study was reviewed and approved by Ben-Gurion University of the Negev.

## Author Contributions

AB designed the research study, performed the research, analyzed the data, and wrote the manuscript. AB and YG interpreted the data. YG and MH conducted a critical revision of the manuscript. All authors contributed to the article and approved the submitted version.

## Conflict of Interest

The authors declare that the research was conducted in the absence of any commercial or financial relationships that could be construed as a potential conflict of interest.

## Abbreviations

HBO: hyperbaric oxygen
HBOT: HBO therapy
HBOTox: hyperbaric oxygen toxicity
HP: high pressure
HPNS: high pressure neurological syndrome.
